# Dose-Responsive Effects of Iron Supplementation on the Gut Microbiota in Middle-Aged Women

**DOI:** 10.3390/nu16060786

**Published:** 2024-03-10

**Authors:** Jane Shearer, Shrushti Shah, Martin J. MacInnis, Grace Shen-Tu, Chunlong Mu

**Affiliations:** 1 Faculty of Kinesiology, University of Calgary, Calgary, AB T2N 1N4, Canada; jshearer@ucalgary.ca (J.S.); shrushti.shah@ucalgary.ca (S.S.); martin.macinnis@ucalgary.ca (M.J.M.); 2 Department of Biochemistry and Molecular Biology, Cumming School of Medicine, University of Calgary, Calgary, AB T2N 1N4, Canada; 3 Alberta Children’s Hospital Research Institute, University of Calgary, Calgary, AB T2N 1N4, Canada; 4 Alberta’s Tomorrow Project, Cancer Control Alberta, Alberta Health Services, Calgary, AB T2T 5C7, Canada; grace.shentu@albertahealthservices.ca

**Keywords:** middle-age, women, gut microbiota, oral iron supplementation, iron deficiency, gut health

## Abstract

Oral iron supplementation is the first-line treatment for addressing iron deficiency, a concern particularly relevant to women who are susceptible to sub-optimal iron levels. Nevertheless, the impact of iron supplementation on the gut microbiota of middle-aged women remains unclear. To investigate the association between iron supplementation and the gut microbiota, healthy females aged 40–65 years (*n* = 56, BMI = 23 ± 2.6 kg/m^2^) were retrospectively analyzed from the Alberta’s Tomorrow Project. Fecal samples along with various lifestyle, diet, and health questionnaires were obtained. The gut microbiota was assessed by 16S rRNA sequencing. Individuals were matched by age and BMI and classified as either taking no iron supplement, a low-dose iron supplement (6–10 mg iron/day), or high-dose iron (>100 mg/day). Compositional and functional analyses of microbiome data in relation to iron supplementation were investigated using various bioinformatics tools. Results revealed that iron supplementation had a dose-dependent effect on microbial communities. Elevated iron intake (>100 mg) was associated with an augmentation of Proteobacteria and a reduction in various taxa, including *Akkermansia*, *Butyricicoccus*, *Verrucomicrobia*, *Ruminococcus*, *Alistipes*, and *Faecalibacterium*. Metagenomic prediction further suggested the upregulation of iron acquisition and siderophore biosynthesis following high iron intake. In conclusion, adequate iron levels are essential for the overall health and wellbeing of women through their various life stages. Our findings offer insights into the complex relationships between iron supplementation and the gut microbiota in middle-aged women and underscore the significance of iron dosage in maintaining optimal gut health.

## 1. Introduction

Iron deficiency is the most common and widespread nutritional disorder in the world [1]. While more common in developing countries, iron deficiencies are also prevalent to varying degrees in industrialized nations [2]. Rates of iron deficiency are much higher in females than males because of a lower average hemoglobin concentration and the regular loss of blood through menstruation [3]. Relevant to the present study, middle-aged women can also be vulnerable to iron deficiency due to menstrual irregularities associated with menopause, fluctuating hormone levels, and the adoption of an unhealthy lifestyle that includes lower physical activity and poor dietary habits [4].

Low iron can have several consequences on an individual’s health. Sub-optimal iron stores are the leading cause of anemia, a condition where there are not enough red blood cells in the body to carry oxygen to tissues. Anemia can cause fatigue, weakness, shortness of breath, dizziness, and pale skin. Accompanying anemia, low iron stores are well documented to impair cognitive function including poor memory, attention, and concentration [5]. The prevention and treatment of sub-optimal iron levels depend on the underlying cause and severity of the deficiency. While a balanced diet adequate in iron-rich foods is essential, diet alone is often insufficient to correct the problem [6]. In such cases, iron supplements are recommended. Most iron supplements contain non-heme iron in the form of an iron salt such as ferrous sulfate, ferrous gluconate, and ferrous fumarate [7,8,9]. The rate of absorption from these sources is generally very poor [10] and is commonly associated with side effects including gastrointestinal distress, nausea, vomiting, and constipation [11]. 

Many women continue to supplement with iron into middle age with large variations in the dose, type, and form of iron administered. The human body possesses an intrinsic regulatory system to prevent iron overload and secondary side effects, including constipation and dyspepsia [12]. Given that only 5–15% of oral iron is absorbed in the small intestine, any surplus unabsorbed iron enters the large intestine, where a dense microbiota colonizes. Excess iron at this site can lead to adverse events such as gastric discomfort, oxidative stress, and mitochondrial damage [13]. Iron also serves as a fundamental nutrient for gut bacteria such as *Escherichia* that can acquire iron via siderophore biosynthesis and secretion [14]. 

The impact of iron supplementation on the gut microbiota is highly variable with the majority of studies to date suggesting that it is harmful to gut homeostasis [15]. In pregnant women, high iron supplementation (>60 mg/day) decreases *Roseburia* and *Ruminococcus* [16] while supplementation (160 mg/day) in iron deficient women of childbearing age restores *Faecalibacterium* abundance [17]. Another study conducted in adults reported participant-specific alterations in the gut microbiota, mostly attributed to the increase of taxa within the Lachnospiraceae family [18]. Some of these discrepant results are likely due to inter- and intra-individual variations [19], as well as differences across physiological life stages, diet, sex, and iron requirements/status. 

The objectives of the present study were to retrospectively examine the associations between iron supplementation and gut microbiota in middle-aged women. This group was selected as there are clear age [20] and sex-specific distinctions between the male and female gut microbiota [21]. Furthermore, the relationship of the female gut microbiota with oral iron supplementation in middle-aged women remains poorly understood. It was hypothesized that oral iron supplementation would exert dose-dependent effects on gut microbiota composition and function.

## 2. Materials and Methods

### 2.1. Participants

**Figure 1:**
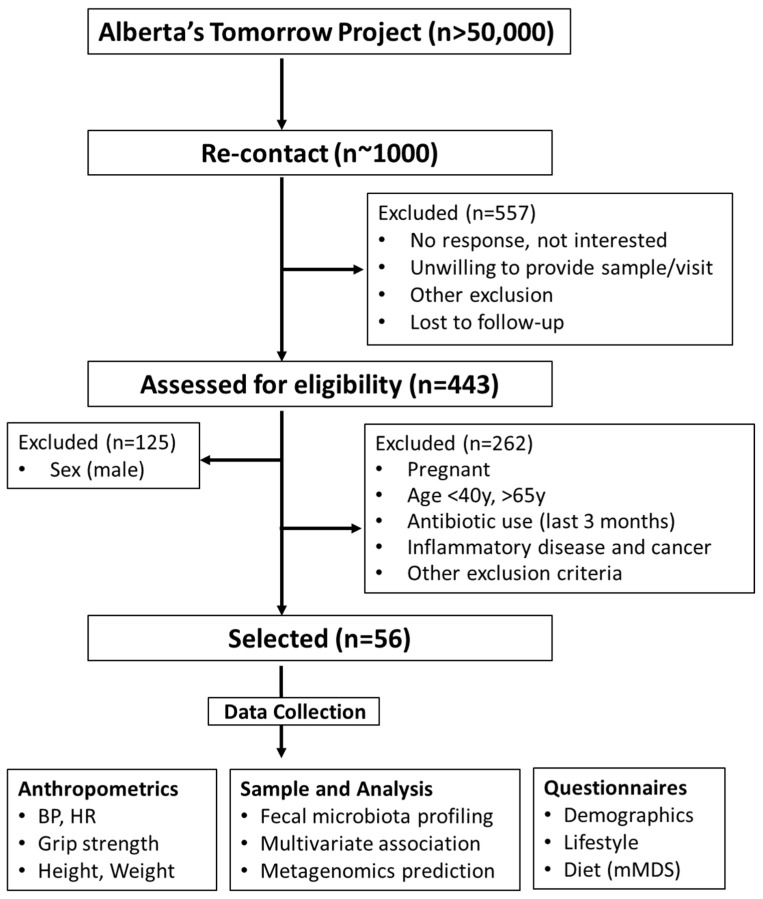
CONSORT flowchart. BMI, body mass index; BP, blood pressure; HR, heart rate; mMDS, modified Mediterranean diet score.

The study was approved by the Conjoint Health Research Ethics Board at the University of Calgary (REB17-1973). All study participants provided written informed consent prior to being enrolled. The study complied with the protocols and clinical practice guidelines of the International Conference on Harmonization and the Declaration of Helsinki. The cohort was a part of Alberta’s Tomorrow Project, a prospective, population-based cohort started in 1999 that has enrolled ~55,000 participants [22,23]. In 2018, a small sample subset of participants (~1000) from the cohort were selected to be re-contacted for the present study. Briefly, participants from the cohort were selected and contacted via phone or email in the Calgary area (Calgary, AB, Canada). A random digit dialing method mapped to Alberta Regional Health Authorities was initially employed to select households with eligible residents. Participants responding to the call and between 35–69 years of age at the time of enrollment were screened for eligibility. In total, 443 individuals (males: 28.2%; females: 71.8%) responded. A CONSORT flowchart is shown in Figure 1.

To study iron–microbiota relationships in middle-age females between 45 and 60 years were retrospectively analyzed based on their iron supplementation status. Individuals were evaluated for suitability according to predetermined inclusion and exclusion criteria. Exclusion criteria included male, pregnant women, those on antibiotics (last 3 months), inflammatory disease, and cancer patients. Eligible participants underwent a battery of measures as previously reported [24]. Among all participants, 23 participants were identified as taking a low-level iron supplement, mainly in the form of a daily women’s specific multivitamin. Dosages in this group ranged from 6–10 mg of iron per day. Another 10 participants were identified as taking a high-dose iron supplement classified as >100 mg/day. These groups were age and body mass index (BMI, kg/m^2^) matched with 23 participants not taking an iron supplement. These groups are herein referred to None-Fe, Low-Fe, and High-Fe, respectively. 

Anthropometric data, including height, weight, waist circumference, resting heart rate, blood pressure, and grip strength, were taken using standard procedures by a trained evaluator within 24 h of stool collection. Additional data, including age, dietary intake, and iron dose, were collected along with medical history, medication use, supplement use, and other lifestyle factors [25]. To assess diet, the Canadian Dietary Health Questionnaire (CDHQ II) was administered to participants [26]. From this, a modified Mediterranean diet score (mMDS) was calculated using a 9-point scale based on food consumption, as previously described [27]. Briefly, diet scores ranged from 0 (least healthy) to 9 (most healthy) and were based on the weekly consumption of 9 food components adjusted by total energy intake: vegetables, legumes, fruits and nuts, dairy, whole grains, meat, fish, alcohol, and fatty acid ratio (calculated as the sum of mono- and poly-unsaturated fatty acids divided by saturated fatty acid intake). A one-way ANOVA and a Tukey’s multiple comparison post hoc test were performed to compare the differences in metadata across the three levels of iron intake. 

### 2.2. Sample Collection, DNA Extraction, and Processing

Stool collection and fecal genomic DNA extraction were performed as described previously [25]. Briefly, stool samples were self-collected using a Protocult^TM^ stool collection device (Ability Building Center, Rochester, NY, USA) and stored at −20 °C immediately after collection. Samples were then delivered to the laboratory, aliquoted, and stored at −80 °C until subsequent DNA extraction. Total fecal genomic DNA was extracted using a QIAamp Fast DNA stool mini kit (Qiagen, Hilden, Germany) as previously described [28]. Blank extraction controls were included for sequencing and statistics. 

### 2.3. High-Throughput Sequencing and Analysis

Library construction and pair-end sequencing were performed following standard protocols using the Illumina MiSeq platform with the MiSeq V3 600 cycle sequencing kit (Illumina, San Diego, CA, USA). Raw sequences were demultiplexed with 0 mismatches in the barcode sequences. Data processing was conducted using the DADA2 version 1.10 workflow to yield amplicon sequence variants (ASVs). ASVs were taxonomically annotated using the Bayesian classifier provided in DADA2 [29] and using the Ribosomal Database Project (RDP) classifier [30]. Data of the ASV table were transformed with centered log-ratio algorithms [31] and analyzed using Microbiome Analyst version 2.0 [32]. Microbial diversity indices, including Good’s coverage rate, Chao1, and Shannon, were calculated to reflect species richness and evenness. Bray–Curtis dissimilarity-based principal coordinates analysis (PCoA) was used to measure differences in microbial structures. Associations between iron dose and microbiota were analyzed with Multivariate Association with Linear Model using *MaAsLin2* [33]. *MaAsLin2* is a comprehensive model that evaluates multivariable associations between clinical metadata and microbial omics features, while correcting for confounding factors including age, BMI, and diet. Considering the exploratory discovery nature of the study, the standard default in *MaAsLin2*, False Discovery Rate (FDR) < 0.25, was used to define statistical significance [34]. 

### 2.4. Metagenomics Prediction of the Microbial Functional Profiles

The potential function of the gut microbiome was predicted from the ASV data using the Tax4Fun metagenomics prediction module [35]. The KEGG Orthologue (KO) data were transformed using centered log-ratio algorithms and imported for partial least squares discriminant analysis (PLS-DA) analysis. Discriminant KOs were determined using one-way ANOVA with Fisher’s Least Significant Difference post hoc test. *p* values from the one-way ANOVA test were adjusted with FDR methods to obtain *q* values (*q* < 0.25). 

## 3. Results

### 3.1. Subject Characteristics

Participant characteristics are shown in Table 1. There were no differences in age (*p* = 0.113), BMI (*p* = 0.580), weight (*p* = 0.388), grip strength (*p* = 0.084), systolic blood pressure (*p* = 0.319), diastolic blood pressure (*p* = 0.345), resting heart rate (*p* = 0.262), or diet score (*p* = 0.207) between the groups. Age, BMI, and diet score were incorporated as the main factors for subsequent data correction. The rate of daily iron supplementation among females in our cohort was 12.6%, inclusive of all doses of iron consumed (Low-Fe, High-Fe).

### 3.2. Microbiota Diversity 

**Figure 2:**
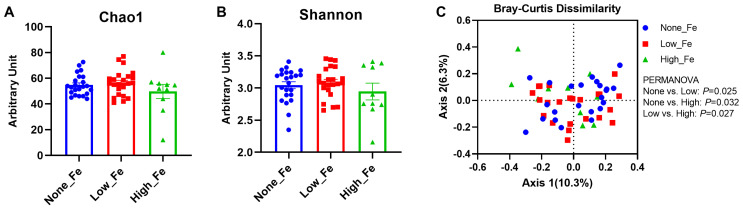
Microbial diversity. (**A**) Chao1 index. (**B**) Shannon index. (**C**) Bray–Curtis dissimilarity-based principal coordinates analysis. The PERMANOVA test was employed to compare the structure of the microbiota between groups. Axis 1 explained 10.3% of the variance and Axis 2 explained 6.3% of the variance. *n* = 23, 23, and 10 for the None-Fe, Low-Fe, and High-Fe groups, respectively.

The 16S rRNA sequencing yielded 1,290,439 reads in total and 23,043 reads on average per sample (SD: 9041 reads) and had a Good’s coverage rate > 99% (99.4–100%), indicating the sequencing depth was sufficient to capture the microbiota diversity [36]. There were no differences in the microbial diversity indices, including Chao1 (Figure 2A) and Shannon evenness (Figure 2B), although high iron intake had the lowest diversity levels when compared to the None-Fe and Low-Fe iron groups (*p* > 0.05). 

When comparing the between-group variation with Bray–Curtis dissimilarity distance, significant differences were found between all groups: None-Fe versus Low-Fe (*p* = 0.025); None-Fe versus High-Fe (*p* = 0.032); Low-Fe versus High-Fe (*p* = 0.027) (Figure 2C). This result indicated that oral iron supplementation had a prominent impact on individual microbial composition rather than overall structural diversity.

### 3.3. Microbiota Composition

**Figure 3:**
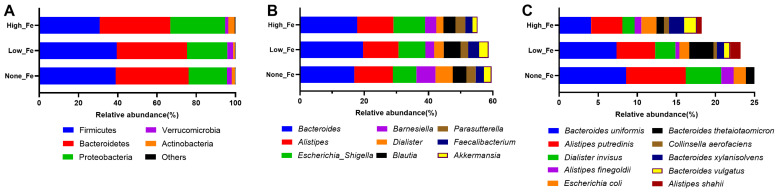
Microbial composition at phylum (**A**), genus (**B**), and species (**C**) levels. Predominant taxa were shown. Data were expressed as relative abundances (percentage of total communities).

At the phylum level, Firmicutes, Bacteroidetes, and Proteobacteria were the dominant taxa that accounted for >90% of the total communities (Figure 3A). The relative abundance of Proteobacteria was higher in High-Fe (28%) than None-Fe (19.6%) and Low-Fe (20.6%). Corresponding to the phylum level composition, *Bacteroides*, *Alistipes*, *Escherichia*/*Shigella*, *Barnesiella*, *Faecalibacterium*, and *Akkermansia* were the dominant taxa at the genus level (Figure 3B). The relative abundances of *Faecalibacterium* and *Akkermansia* were lower in High-Fe than the None-Fe and Low-Fe groups. At the species level, *Bacteroides uniformis*, *Alistipes putredinis*, *Escherichia coli*, and *Bacteroides thetaiotaomicron* were among the dominant taxa (Figure 3C).

**Figure 4:**
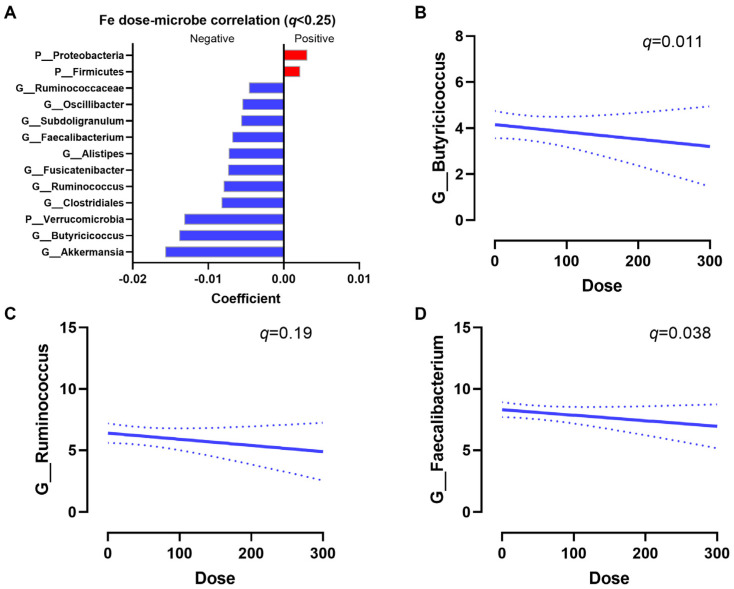
Iron dose–microbiota associations. (**A**) Significant associations between bacteria taxa and iron dose. Positive correlations indicate that a high iron dose increases bacterial abundances, while a negative correlation indicates the high iron dose decreases bacterial abundances. Significance was defined at *q* < 0.25 by *MaAsLin2*. Representative regression plots of *Butyricicoccus* (*q* = 0.011) (**B**), *Ruminococcus* (*q* = 0.19) (**C**), and *Faecalibacterium* (*q* = 0.038) (**D**) with iron dose are shown. Dashed lines indicate the 95% confidence interval.

To identify associations between iron dose and bacterial taxa, multivariate regression analyses were performed. After correcting for age, BMI, and diet score, Proteobacteria and Firmicutes were positively associated with an increased iron intake, while *Akkermansia*, *Butyricicoccus*, *Verrucomicrobia*, *Ruminococcus*, *Alistipes*, and *Faecalibacterium* were negatively associated with an increased iron intake (*q* < 0.25, Figure 4A). The regression slopes of *Butyricicoccus* (*q* = 0.011), *Ruminococcus* (*q* = 0.19), and *Faecalibacterium* (*q* = 0.038) are shown (Figure 4B–D). Increasing iron supplementation also had a trend to be positively associated with *Escherichia*/*Shigella* (raw *p* = 0.1). Overall, increases in oral iron supplementation had a strong impact on the microbial composition.

### 3.4. Predicted Metagenomics Function

**Figure 5:**
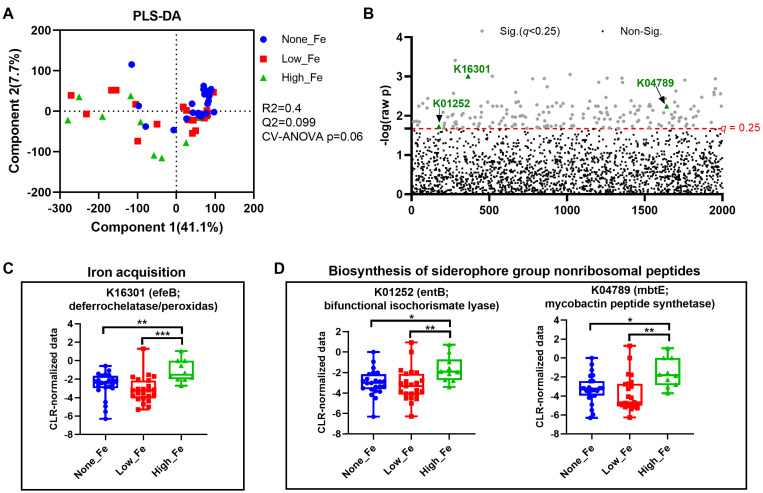
Metagenomics prediction of microbial functions. (**A**) PLS-DA scatter plot. (**B**) Alterations of KEGG Orthologues (KOs). (**C**) Alteration of K16301, an enzyme responsible for iron acquisition. (**D**) Alteration of KOs involved in siderophore biosynthesis. * *p* < 0.05, ** *p* < 0.01, *** *p* < 0.001 by one-way ANOVA with Fisher’s Least Significant Difference post hoc test.

To understand how oral iron supplementation affects microbial functional potential, metagenomics prediction was performed with Tax4Fun [35]. PLS-DA modelling showed a trend toward separation (*p* = 0.06) (Figure 5A) with the None-Fe group appearing to have closer clustering compared to those consuming an iron supplement. One-way ANOVA identified a total of 177 KEGG Orthologues that differed between groups (Figure 5B). Among the discriminant KOs, the relative abundance of K16301 that encodes an enzyme for iron acquisition was higher in the High-Fe group than the None-Fe and Low-Fe groups (*p* < 0.01, Figure 5C). Additionally, the relative abundances of two KOs, K01252 and K04789 that encode enzymes for siderophore function were also higher in High-Fe than None-Fe and Low-Fe (*p* < 0.05, Figure 5D). Overall, oral iron supplementation potentially affected microbial function, particularly those involved in iron acquisition. 

## 4. Discussion

Sufficient iron intake plays a crucial role in the overall health and wellbeing of females at all life stages. Although often overlooked, many middle-aged women are susceptible to iron-related deficiencies requiring iron supplementation. Employing a healthy female cohort, this data provides valuable insights into the impact of iron supplementation on the gut microbiota. Novel results show that even low doses of iron (6–10 mg/day), an amount found in many female-targeted multivitamins, influences the gut microbiota and that there is a dose–response relationship between iron supplementation and the gut microbiota. Supplementing with high doses of iron (>100 mg/day) was linked to alterations in the gut microbiota, marked by a decrease in certain beneficial bacterial strains associated with gastrointestinal health, alongside a concurrent rise in those associated with inflammatory reactions.

The human microbiota has a diverse array of metabolic idiosyncrasies related to nutritional preference [37]. This was evident in the present study with certain genera and species of bacteria changing in response to both low and high-dose iron supplementation. Using multivariate analysis controlled for age, BMI, and diet, we identified several iron dose–microbiota associations of biological relevance. Notably, the observed increase of Proteobacteria may represent a microbial signature associated with high iron supplementation. In this study, microbes of Proteobacteria such as Enterobacteriaceae and *Escherichia*/*Shigella* appear to be quite sensitive to iron supplementation. Iron depletion can reduce *Esherichia* species in batch culture of human feces [38], while iron-sufficient conditions could quickly restore *Escherichia fergusonii* [18]. Studies in Kenyan infants show that six months of iron fortification increases *Escherichia*/*Shigella* and pathogenic *Escherichia coli*, changes that were accompanied by an elevation in intestinal inflammation [39,40]. Therefore, an expansion in the Proteobacteria species could serve as a biomarker indicative of a proinflammatory response after high iron supplementation, although more work is needed to confirm this observation.

Like humans, iron is essential to bacterial metabolism and microbes have developed complex systems to compete for the metal [41]. There are three general strategies that bacteria use to acquire iron which are as follows: using ferric-specific chelators known as siderophores, using host iron-containing compounds including heme and transferrin, and absorbing ferrous iron following reduction [42]. In the present study, we show an upregulation of predicted siderophore function with high iron supplementation. This finding is consistent with earlier studies showing high iron intake to enrich siderophore biosynthesis using metagenomics prediction in women consuming >65 mg of iron daily [16]. Although we are unable to identify which microbes are responsible for the increased siderophore functions, it is highly likely this is due to an upregulation of *Escherichia*/*Shigella* that contain microbes with high iron demands [43]. Since metagenomics predictions based on 16S rRNA data can only infer potential functional capabilities, future studies using direct metagenomic sequencing [44] would provide more definitive insights into the functional implications of iron supplementation on the gut microbiome.

In the present study, increasing iron dose had negative associations with several microbial taxa, including *Akkermansia*, *Butyricicoccus*, *Ruminococcus*, and *Faecalibacterium*. The clinical consequences of these alterations remain unknown. *Akkermansia* species are typical mucin-degrading bacteria with proven benefits in maintaining gut barrier function, short-chain fatty acid production, and in counteracting intestinal inflammation [45,46]. Decreases of *Akkermansia* have also been observed in specific-pathogen-free mice fed a high iron diet [47] and in colorectal cancer mice with excessive iron intake [48]. In addition to *Akkermansia*, species of *Faecalibacterium* also colonize the mucus layer. Since *Akkermansia* relies on intestinal mucin to survive [49], it is of interest to consider whether high iron supplementation affects mucin synthesis, a process that is tightly related to gut barrier function.

Another finding of interest in our data was a reduction in *Ruminococcus* with iron supplementation that appeared to be dose-dependent. This finding fits with early reports that *Ruminococcus* tends to be reduced in women supplemented with up to 65 mg/d iron in early pregnancy [16]. The effects of iron supplementation on the gut microbiota are garnering increased interest due to the complex microbial interactions involved. We observed that *Ruminococcus*, *Faecalibacterium*, and *Akkermansia* declined with increasing iron supplementation. Microbes within the *Ruminococcus* genus, particularly the keystone species *Ruminococcus bromii* [50,51], play a crucial role in degrading resistant starch. They facilitate the release of simple carbohydrates, that can be utilized downstream by *Faecalibacterium* species to produce butyrate [52] or by *Akkermansia* species to produce propionate [53]. These examples illustrate the role of individual taxa in forming a metabolic chain that sustains energy fuels for colonocytes and maintains gut homeostasis [54]. Given this, decreases in the abundances of *Ruminococcus*, *Faecalibacterium*, and *Akkermansia* following high iron supplementation may indicate alterations in metabolic cross-feeding activities, potentially affecting the gut microbiota’s ability to utilize carbohydrates. It is deduced that high iron may adversely affect microbial fermentation and that this impact is likely related to an increased occurrence of constipation following high-iron supplementation [55].

As with any retrospective observational investigation, there are several study strengths and limitations that need to be considered. Key strengths of this study include the strict control of confounding factors including age, BMI, and diet. This study was limited by the relative shortage of middle-aged female participants consuming high iron (>100 mg/d) within our cohort of 443 individuals. This limitation can be overcome by expanding the cohort in the future and/or considering those individuals on alternate iron supplementation regimens (e.g., oral iron supplementation every second day). Due to the limited cohort size, we are unable to distinguish the impact of supplement duration as well as different forms of iron supplementation on the gut microbiota. It is very likely that different iron forms, enhancers (e.g., ascorbic acid), and inhibitors (e.g., calcium), as well as dietary components such as fiber, also affect the gut microbiota and iron absorption [56]. Furthermore, there is growing interest in exploring the personalized aspects of iron supplementation, as highlighted by the concept of precision nutrition [57]. This is supported by recent findings showing individualized changes in the gut microbiota following ferrous sulfate supplementation in adults [18]. These differences are not only influenced by genetics [44], but also variation in daily routines including sleep and exercise [24,58]. Understanding how such factors impact the gut microbial response to iron supplementation requires further investigation.

## 5. Conclusions

These results provide evidence that high iron intake (>100 mg/d) is associated with alterations in the composition of the gut microbiota in healthy, middle-aged females. Moving forward, recommendations to supplement with high-dose iron should be considered in light of its adverse impacts on the gut microbiota and its potential to promote gastrointestinal inflammation.

## Figures and Tables

**Table 1:** Participant characteristics.

	None-Fe (*n* = 23)	Low-Fe (*n* = 23)	High-Fe (*n* = 10)	*p*-Value
Age (years)	54.8 ± 7.4	57.2 ± 5.8	51.7 ± 7.2	0.113
BMI (kg/m^2^)	23.2 ± 3.3	23.6 ± 2.4	23.1 ± 1.3	0.580
Weight (kg)	63.7 ± 7.6	63.4 ± 8.7	62.5 ± 3.3	0.388
Grip strength (kg)	29.3 ± 4.5	27.9 ± 4.4	32.7 ± 6.5	0.084
SBP (mmHg)	115.2 ± 14.9	110.4 ± 11.3	109.5 ± 7.0	0.319
DBP (mmHg)	71.8 ± 9.1	68.9 ± 8.7	67.8 ± 4.2	0.345
Heart rate (bpm)	66.5 ± 8.3	62.4 ± 8.1	65.4 ± 9.5	0.262
Diet score (mMDS)	4.9 ± 1.8	4.3 ± 1.5	3.9 ± 1.8	0.207

Data is presented as mean ± SD. BMI, body mass index; DBP, diastolic blood pressure; mMDS, modified Mediterranean diet score (0–9); SBP, systolic blood pressure. One-way ANOVA and Tukey’s multiple comparison test were performed. Statistical significance was set at *p* < 0.05.

## Data Availability

Sequencing data have been deposited in NCBI’s Sequence Read Archive (SRA) database under accession number PRJNA 922681.
